# Reliability and validity of Foot Posture Index (FPI-6) for evaluating foot posture in participants with low back pain

**DOI:** 10.1038/s41598-022-22220-1

**Published:** 2022-12-07

**Authors:** Jiaman Yang, Zhiwen Ou, Zhitao Mao, Yi Wang, Yiheng Zhong, Wei Dong, Zhen Shen, Zehua Chen

**Affiliations:** 1 The Fifth Clinical College of Guangzhou, University of Chinese Medicine, Guangzhou, China; 2 The orthopedics hospital of traditional Chinese medicine Zhuzhou city, Zhuzhou, China; 3 Department of Orthopaedics, Kunming Municipal Hospital of Traditional Chinese Medicine, The Third Affiliated Hospital of Yunnan University of Chinese Medicine, Kunming, China

**Keywords:** Medical research, Preclinical research

## Abstract

Previous studies have demonstrated that Foot Posture Index (FPI-6) is a valid and moderately reliable tool to evaluate foot posture. However, data about reliability and validity of FPI-6 in the assessment of foot posture in people with low back pain (LBP) is lacking. To investigate reliability and validity of FPI-6 in the assessment of foot posture in people with LBP. Thirty volunteers with LBP, aged 20–64 years, were recruited for the research and assessed by two raters. The data measured by different raters on the same day were used to calculate the inter-rater reliability. The data measured by the same rater on different dates were used to calculate the test–retest reliability. The reliability of FPI-6 was tested with intraclass correlation coefficient (ICC), and absolute reliability with standard error of measurement (SEM), minimal detectable change (MDC) and Bland–Altman analysis. The validity of FPI-6 was tested with Exploratory Factor Analysis (EFA) and Spearman's correlation coefficients. The FPI-6 indicated excellent inter-rater and test–retest reliability in the evaluation of foot posture in people with LBP (ICC = 0.97 and 0.95). The agreement for inter-rater and test–retest was excellent based on the SEM (SEM = 0.12) and MDC value (MDC = 0.33). Bland–Altman plots showed that there was no significant systematic bias for the agreement on the ground of low mean difference (< 1). The EFA suggested that the fit indices were considered acceptable according to the Kaiser–Meyer–Olkin (KMO) value (KMO = 0.620) and Bartlett's sphericity test (*P* < 0.01). There was a statistically significant positive correlation between each item and total score of FPI-6 because the Spearman’s correlation coefficient of six items were all > 0.3 (*P* < 0.01). The inter-rater and test–retest reliability and validity of FPI-6 on people with LBP were proved reliable. It might be considered a reliable and valid adjunctive tool to detect possible changes of foot posture after interventions in patients with LBP.

## Introduction

Low back pain (LBP) is the most prevalent condition in most countries, and the number of people disabled by LBP is rapidly increasing^1^. Those disabled by LBP mainly involves dysfunction of activity limitations^2^. As well as activity limitations, pain, musculoskeletal complaint and postural balance dysfunction are necessary to be addressed in the treatment of LBP patients in order to prevent afflicting quality of life and work^3^. In fact, LBP is the leading health condition contributing to the need for rehabilitation services and work absenteeism in most countries, especially in low-income and middle-income countries, causing a significant financial burden for affected individuals and society^4^. Whereas, given that multiple factors encompassing psychological, biophysical, genetic and social factors result in the development and maintenance of LBP, the LBP management is still a great challenge for global clinicians^5,6^.

In addition to the above pathogenic factors, it has been believed that abnormal foot posture could also be a contributing factor^7,8^. Furthermore, from the biomechanical and physiology perspective, it has been widely recognized that the association between foot posture and LBP is plausible^9–11^. The normal foot posture provides stability for limb^9^. However, abnormal foot posture has been demonstrated to trigger abnormal posture of the femur and tibia, leg length discrepancy, and may consequently cause anterior inclination of the pelvis and abnormal position of the lumbar vertebrae^11^. Moreover, there is evidence that foot orthoses can alleviate LBP^10,11^. We can think that the alteration of foot position can cause disturbance of joint biomechanics in the low back thereby affecting pelvic alignment and normal position of the spine and eventually leading to LBP^12^. Thus, the assessment of foot posture could be important for understanding the pathogenesis of LBP and may be an important part of treatment planning that cannot be ignored^13^.

Indeed, there is growing interest in investigating the association between the foot posture and LBP, from a biomechanical perspective. The device of motion analyser^7^ and the MatScan system^14^ were used to investigate the foot posture and function in previous studies. Nevertheless, not only are these devices very expensive, but they are not easily available to all clinicians. In contrast, (FPI-6) is considered a novel clinical tool to observe and measure foot posture features quickly and easily. FPI-6 can provide detailed quantitative data of foot postural variation^15^. And it has been proved to be the most relevant for evaluating foot posture including six evaluation criteria founded on three-dimensional observations^16^. It just takes clinicians approximately 5 min to complete the assessment without any special device^17^. Additionally, the FPI-6 was designed to properly provide actual foot postural variations in the region of forefoot, rear foot, and midfoot compared with other instruments (e.g., three-dimensional static model during weight bearing activity). More importantly, it can be carried out almost everywhere and particularly applied for studies where complex instrumented assessment is unnecessary^18^. Previous studies have provided evidence for the validity of FPI-6 as a clinical tool, as demonstrated excellent intra -rater reliability and low to moderate inter-rater reliability^15,19^.

To summarize, studies on validity and reliability of FPI-6 as a clinical tool to assess foot posture features have shown encouraging results. But data on the inter-rater and test–retest reliability and validity on people with LBP is lacking. To our knowledge, studies assessing reliability and validity of FPI-6 in people with LBP have not been published to date. Thus, the aim of this study was to investigate the inter-rater and test–retest reliability and validity of FPI-6 on people with LBP to provide evidence for the application of FPI-6 in clinical practice.

## Methods

### Setting

Measurements were performed at the outpatient clinic of Guangdong Second Traditional Chinese Medicine Hospital in China. All volunteers with LBP signed written informed consent. The study protocol was approved by the Ethics Committee of Guangdong Second Traditional Chinese Medicine Hospital (No. 2021(K69)) and registered in Chinese Clinical Trial Registry (registration number: ChiCTR2200055265, registration date: 05/01/2022). All methods were performed in accordance with relevant guidelines and regulations. Additionally, we promised to provide a free treatment for volunteers when the study finished.

### Participants

Zou GY^20^ present a method to estimate the number of participants required in reliability studies. This method explicitly incorporates a prespecified probability of achieving the prespecified width or lower limit of a confidence interval. The hypothesis was that we wish to ensure, with 80% probability, that the lower limit of the one-sided 95% confidence interval is no less than 0.7 when the anticipated value of intraclass correlation coefficient is 0.725. Then, the number of participants we need is 16 according to equation. In this study, a sample size of 30 participants was estimated. The inclusion criteria for all participants were: (1) persistent low back pain greater than 3 months with pain intensity at least 4 on the numerical rating scale; (2) aged 20–64 years (3) being able to remain in static orthostatic position; (4) no history of surgery or injury to the back or lower limbs; (5) no structural deformities of the lower limbs. The exclusion criteria were: (1) injuries to the back or lower limbs (i.e., musculoskeletal injuries, foot deformities) in previous 6 months or during the assessments; (2) surgery to back or lower extremity previously or currently under treatment for foot pathology.

### Procedure

All participants were independently assessed by two raters (J Y and Z M) in order to assess inter-rater and test–retest reliability. Two raters participated in a training session for familiarization with the clinical tool. They communicated with each other during the training period for familiarizing each item of the FPI-6 before the first measurement. After finishing the training session, they completed 40 measurement of FPI-6 before using the values for further analysis of this study according to the recommendations of Cornwall et al.^21^. And the data collected was not used, which is just for familiarization with the assessment procedure and minimizing inter-rater error. Each item of FPI-6 was independently assessed and scored by each rater in separate sheets. Participants were asked to stand, take a few steps forward and then stand still with arms along the side and looking forward. The first test was performed by one rater (J Y), who evaluated the left foot first and then the right foot. Six items of the FPI-6 were all assessed: (1) talar head palpation, (2) observation of curves above and below the lateral malleolus, (3) a bulge in the region of the talonavicular joint, (4) eversion and inversion of the calcaneus, (5) congruence of the medial longitudinal arch, (6) adduction and abduction of the forefoot in relation to the rear foot. The score for each item was rated between − 2 and + 2, and the total score was between − 12 and + 12. Scores between 0 and + 5 indicate normal feet; + 6 to + 9 indicate pronated feet; ≥  + 10 indicate highly pronated feet; − 1 to − 4 indicate supinated feet; − 5 to − 12 indicate highly supinated^22^. When the first rater (J Y) finished the assessment, the participants remained in their positions and were assessed by the second rater (Z M). The data measured by different raters on the same day were used to calculate the inter-rater reliability. Then a second test was performed by rater (J Y) for each participant approximately one week apart. The data measured by the same rater (J Y) on different dates were used to calculate the test–retest reliability. The second rater (Z M) was blinded to the rater (J Y) and to their own results during the assessment. Additionally, both feet of all participants were considered for analysis.

### Statistical analysis

All data were imported and analysed using the SPSS 24.0 for Windows (IBM, NY, US). The normality was assessed firstly with collected data using the Shapiro-Wilks test. Almost all data were not normally distributed therefore non-parametric statistics were used. Inter-rater and test–retest reliability were assessed using Intra-class Correlation Coefficients (ICCs). ICC is a desirable reliability index that can be used to reflect both degree of correlation and agreement between measurements. Nowadays, it has been widely used to evaluate inter-rater and test–retest reliability of numerical or continuous measurements. Inter-rater reliability was assessed using two-way random model, mean measures. And test–retest reliability was assessed using two-way mixed model, single measures^23^. The ICC value ranges between 0 and 1, with the values closer to 1 indicating higher reliability. The reliability was considered poor when the ICC < 0.4, fair when the ICC ≥ 0.4 –  ≤ 0.59, good when the ICC ≥ 0.6–≤ 0.74 and excellent when the ICC ≥ 0.75^24^. The absolute reliability was assessed using standard error of measurement (SEM, SEM = standard deviation × $\surd$1 − ICC), minimal detectable change (MDC, MDC = 1.96 ×  SEM × $\surd$2), and 95% limits of agreement (LOA)^25^. Bland–Altman plots was used by the Medcalc software version 20.0 (Medcalc, Ostend, Belgium) to assess the agreement and identify systematic bias for inter-rater^26^. Exploratory Factor Analysis (EFA) based on principal component analysis with the varimax rotation was conducted to assess the validity. The purpose of this method was to represent the original data structure with fewer data dimensionality and explain the correlations between each item with fewer underlying factors^27^. First the Kaiser–Meyer–Olkin (KMO) was used to evaluate the correlation of the variables. KMO value is between 0 and 1. The larger of KMO value, the higher the correlation of the variables^28^. It was suitable for factor analysis when the KMO value > 0.6^29^. Then a Bartlett sphericity test was used as a verification tool to further confirm that the correlation matrix was an identity matrix. It was suitable for factor analysis when the *P* value < 0.05^30^. Additionally, the correlation between each item and the total scores was assessed to complement the factor analysis using Spearman’s correlation coefficient. The validity was acceptable when the correlation coefficients > 0.30^31^. Statistical significance was defined as *P* < 0.05.

## Results

### Participants characteristics

Thirty participants (16 women and 14 men) with LBP were included in this study. The mean age of all participants was 42.47 ± 6.89 years, mean height was 164.27 ± 8.61 cm, mean weight was 62.17 ± 9.74 kg, mean VAS was 6.03 ± 1.40 and BMI was 22.94 ± 2.00 kg/m^2^. The characteristics of all included participants were shown in Table 1.

**Table 1:** A summary of the characteristics of all participants.

Variable	Mean ± SD
Age (years)	42.47 ± 6.89
Height (cm)	164.27 ± 8.61
Weight (kg)	62.17 ± 9.74
BMI (kg/m^2^)	22.94 ± 2.00
NRS	6.03 ± 1.40

BMI, body mass index; NRS, numerical rating scale.

Homogeneity of continuous data for each leg was analyzed to determine whether single-leg data could be pooled. The ICC of left limb, right limb and left and right limbs was 0.97 (95% CI 0.95–0.99), 0.97 (95% CI 0.94–0.98) and 0.96 (95% CI 0.94–0.98), respectively. From this analysis, the data were suitable to be pooled with little differences between the left and right limb^32^. Pooling had the effect of doubling the sample size, that is, data could reasonably be analyzed on the basis of individual limb rather than participant numbers. Hence, after this analysis, the study included 30 participants and 60 limbs.

### Inter-rater reliability

The results of inter-rater reliability of FPI-6 were shown in Table 2. The ICC of FPI-6 total score was 0.97 (95% CI 0.95–0.98). And ICC values of six items were all > 0.75. The results demonstrated that the inter-rater reliability of FPI-6 was excellent. In addition, the ICC value of Item 2 was the lowest compared with the other five items.

**Table 2:** Inter-rater reliability of the FPI-6.

Variable	ICC	95% CI	*P* value
Total FPI-6	0.97	0.95–0.98	< 0.01
Item 1	0.88	0.80–0.93	< 0.01
Item 2	0.80	0.67–0.88	< 0.01
Item 3	0.89	0.82–0.94	< 0.01
Item 4	0.85	0.76–0.91	< 0.01
Item 5	0.89	0.81–0.93	< 0.01
Item 6	0.90	0.83–0.94	< 0.01

Total FPI-6, Foot total score of FPI-6; ICC, intraclass correlation coefficient; Item 1, talar head palpation; Item 2, curves above and below the lateral malleolus; Item 3, a bulge in the region of the talonavicular join; Item 4, eversion and inversion of the calcaneus; Item 5, congruence of the medial longitudinal arch; Item 6, adduction and abduction of the forefoot in relation to the rear foot.

### Test–retest reliability

The results of test–retest reliability of FPI-6 were shown in Table 3. The ICC of FPI-6 total score was 0.95 (95% CI 0.92–0.97). And ICC values of six items were all > 0.75. The results demonstrated that the test–retest reliability of FPI-6 was excellent. In addition, the ICC value of Item 5 was the lowest compared with the other five items.

**Table 3:** Test–retest reliability of the FPI-6.

Variable	ICC	95% CI	*P* value
Total FPI-6	0.95	0.92–0.97	< 0.01
Item 1	0.83	0.71–0.90	< 0.01
Item 2	0.87	0.78–0.92	< 0.01
Item 3	0.90	0.83–0.94	< 0.01
Item 4	0.88	0.80–0.93	< 0.01
Item 5	0.80	0.66–0.88	< 0.01
Item 6	0.82	0.70–0.89	< 0.01

Total FPI-6, Foot total score of FPI-6; ICC, intraclass correlation coefficient’ Item 1, talar head palpation; Item 2, curves above and below the lateral malleolus; Item 3, a bulge in the region of the talonavicular join; Item 4, eversion and inversion of the calcaneus; Item 5, congruence of the medial longitudinal arch; Item 6, adduction and abduction of the forefoot in relation to the rear foot.

### Absolute reliability

The results of SEM, MDC, and 95% LOA were shown in Table 4. The SEM value of FPI-6 total score for inter-rater and test–retest was 0.17 and the MDC value of FPI-6 total score for inter-rater and test–retest was 0.47. Additionally, the SEM value of all six items for inter-rater and test–retest ranged from 0.03 to 0.05 and the MDC of all six items for inter-rater and test–retest ranged from 0.08 to 0.14.

**Table 4:** The SEM, MDC and 95% LOA of inter-rater and test–retest.

Variable	Inter-rater					Test–retest				
	Mean	SEM	MDC	95% LOA		Mean	SEM	MDC	95% LOA	
				Lower	Upper				Lower	Upper
Total FPI-6	7.27	0.17	0.47	− 1.09	1.29	7.31	0.17	0.47	− 1.33	1.39
Item 1	1.36	0.05	0.14	− 0.55	0.68	1.36	0.05	0.14	− 0.67	0.84
Item 2	1.23	0.05	0.14	− 0.73	0.87	1.21	0.05	0.14	− 0.58	0.74
Item 3	1.00	0.05	0.14	− 0.68	0.54	1.00	0.05	0.14	− 0.68	0.54
Item 4	1.33	0.04	0.11	− 0.52	0.62	1.33	0.04	0.11	− 0.48	0.54
Item 5	1.06	0.03	0.08	− 0.46	0.42	1.07	0.04	0.11	− 0.66	0.60
Item 6	1.30	0.05	0.14	− 0.63	0.63	1.30	0.05	0.14	− 0.70	0.66

Total FPI-6, Foot total score of FPI-6; LOA, Limit of Agreement; MDC, minimal detectable change; SEM, standard error of measurement; Item 1, talar head palpation; Item 2, curves above and below the lateral malleolus; Item 3, a bulge in the region of the talonavicular join; Item 4, eversion and inversion of the calcaneus; Item 5, congruence of the medial longitudinal arch; Item 6, adduction and abduction of the forefoot in relation to the rear foot.

**Figure 1:**
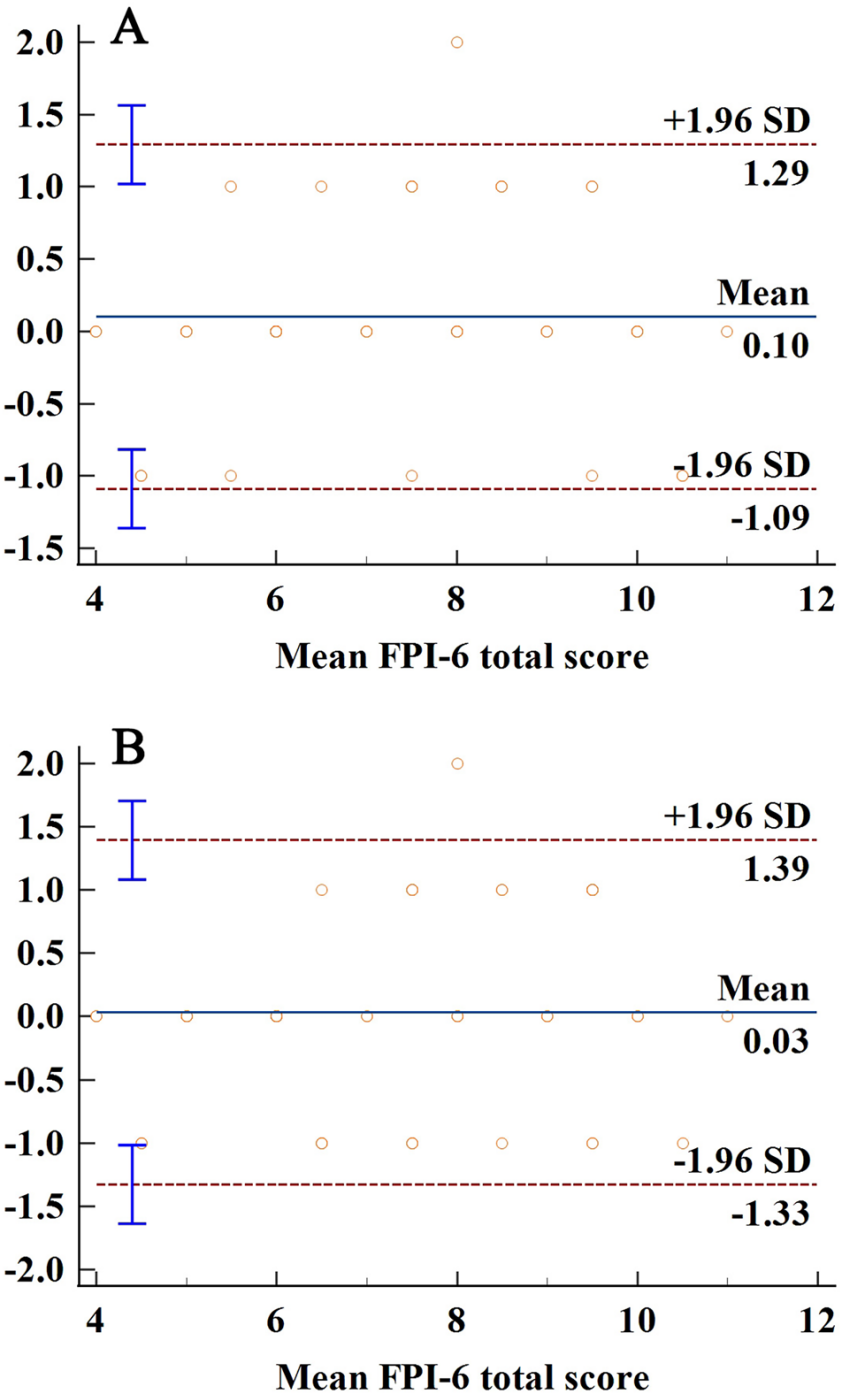
Bland–Altman plots of FPI-6 total score for inter-rater (**A**) and test–retest (**B**).

Additionally, the Bland–Altman graphs with the mean difference and 95% limits of agreement for the level of agreement were showed in Fig. 1. The mean difference of FPI-6 total score for inter-rater and test–retest was 0.10 and 0.03, with the lower and upper limits of − 1.09 to 1.29 and − 1.33 to 1.39, respectively. There was little systematic bias of the FPI-6 and the agreement for inter-rater and test–retest was excellent.

### Validity

**Figure 2:**
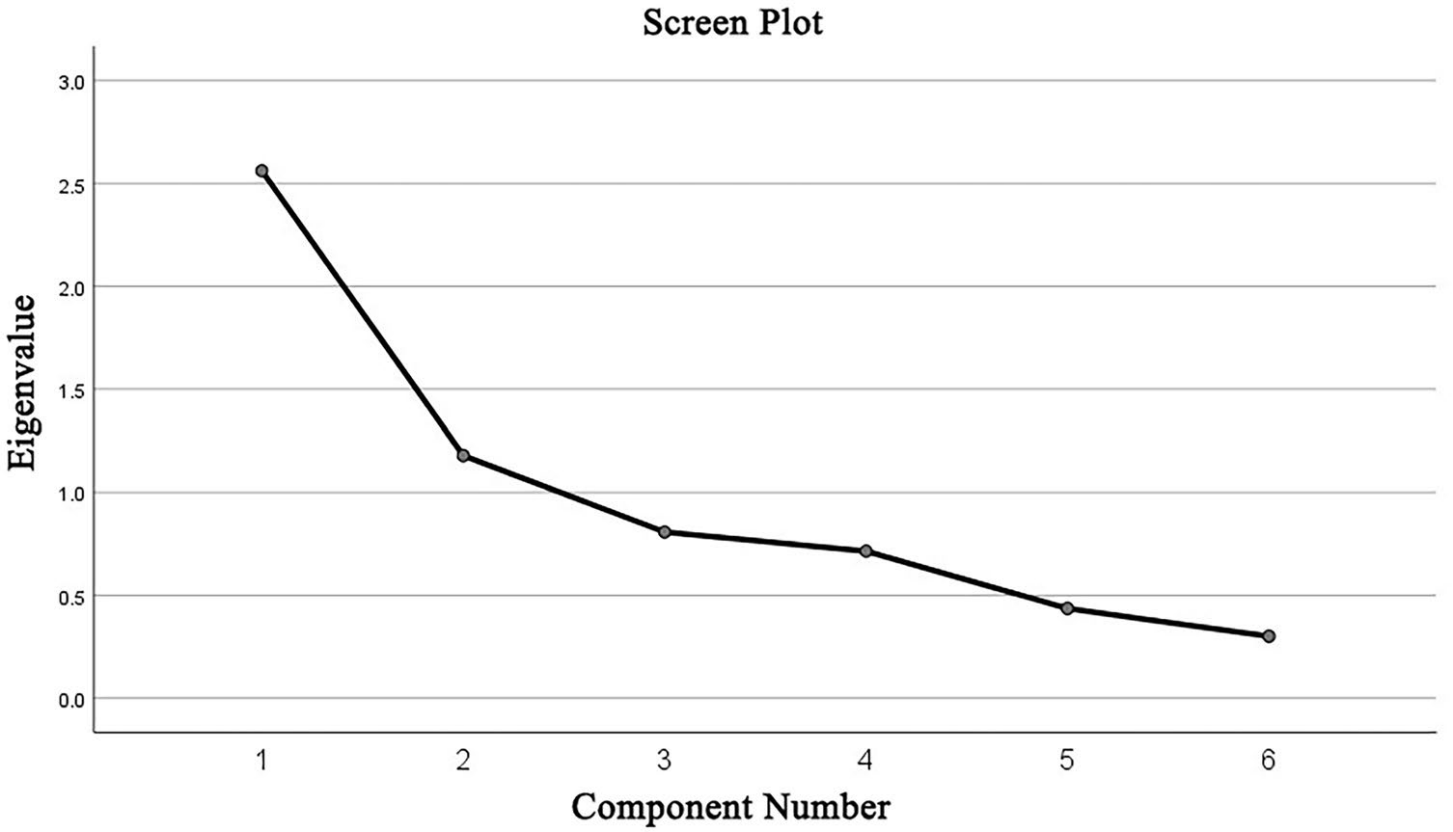
The screen plot of FPI-6.

The value of KMO test was 0.62 (> 0.6) and the Bartlett’s sphericity test (*P* value < 0.01) indicated that the correlation matrix was an identity matrix. This indicates that the EFA was suitable for conducting. In the principal component analysis and the varimax rotation scheme, 2 factors were retained based on the screen plot (Fig. 2) and the Kaiser–Guttman criterion with eigenvalues greaer than 1^33^.

The 6 items were distributed into 2 factors and the highest score in the factors was revealed according to the method of the extraction and rotation. The variable distribution of factorial loading for each of the 2 factors was shown in Table 5. The correlation between each item and total score of FPI-6 in the evaluation of LBP was showed in Table 6. The Spearman’s correlation coefficient of six items were all > 0.3 (*P* value < 0.01). Therefore, there was a statistically significant positive correlation between each item and total score of FPI-6.

**Table 5:** Factor loadings from EFA.

Items	Factors	
	I	II
1. Talar head palpation	0.85	
2. Curves above and below the lateral malleolus	0.80	
3. A bulge in the region of the talonavicular join		0.84
4. Eversion and inversion of the calcaneus	0.65	
5. Congruence of the medial longitudinal arch		0.83
6. Adduction and abduction of the forefoot in relation to the rear foot	0.70

**Table 6:** The correlations between each item and total score of FPI-6.

Variable	Spearman’s correlation coefficient	*P* value
Item 1	0.66	< 0.01
Item 2	0.46	< 0.01
Item 3	0.42	< 0.01
Item 4	0.60	< 0.01
Item 5	0.46	< 0.01
Item 6	0.62	< 0.01

Total FPI-6, Foot total score of FPI-6; LOA, Limit of Agreement; MDC, minimal detectable change; SEM, standard error of measurement; Item 1, talar head palpation; Item 2, curves above and below the lateral malleolus; Item 3, a bulge in the region of the talonavicular join; Item 4, eversion and inversion of the calcaneus; Item 5, congruence of the medial longitudinal arch; Item 6, adduction and abduction of the forefoot in relation to the rear foot.

### Classification of the feet postures

The classification of the feet postures in the three moments of assessment was showed in Table 7. Sixty limbs of 30 participants were assessed at three different times. In the first assessment conducted by rater A (J Y), 41 feet were pronated, 10 as highly pronated, 9 as normal. None of the feet was classified as supinated or highly supinated. In the second assessment conducted by rater B (Z M), 43 feet were pronated, 8 as highly pronated, 9 as normal. None of the feet was classified as supinated or highly supinated. In the third assessment conducted by rater C (J Y) approximately one week apart, 43 feet were pronated, 8 as highly pronated, 9 as normal. None of the feet was classified as supinated or highly supinated. There was no difference in classification of feet postures for inter-rater and test–retest (a chi-square test *P*-value = 0.87).

**Table 7:** Classification of the feet postures in the three moments of assessment.

Rater	*n*	Feet posture					*χ*^2^ value	*P* value
		Pronated	Highly pronated	Normal	Supinated	Highly supinated		
A	60	41	10	9	0	0	0.27	0.87
B	60	43	8	9	0	0		
C	60	43	8	9	0	0	0.27	0.87

A, rater (J Y); B, rater (Z M); C, rater (J Y) approximately one week apart.

## Discussion

To our knowledge, this study represented the first to evaluate reliability and validity of FPI-6 in the assessment of foot posture in people with LBP. In this study, inter-rater, test–retest reliability and validity were assessed in 30 participants with LBP. The results showed excellent inter-rater, test–retest reliability and great absolute agreement of the FPI-6 for inter-rater and test–retest. Moreover, there was a statistically significant positive correlation between each item and total score of FPI-6. Therefore, the FPI-6 was a valid clinical tool that could be used to assess foot posture in people with LBP.

Before any measurement instruments or assessment tools can be used for research or clinical applications, their reliability must be established. Reliability is defined as the extent to which measurements can be replicated. ICC and Bland–Altman plot have been used to evaluate reliability. ICC has been widely used in medicine to evaluate interrater and test–retest reliability. These evaluations are fundamental to clinical assessment, without them, we have no confidence in our measurements, nor can we draw any rational conclusions from our measurements. Inter-rater reliability reflects the variation between 2 or more raters who measure the same group of subjects. Test–retest reliability reflects the variation in measurements taken by an instrument on the same subject under the same conditions. Additionally, validity is about the closeness of what we believe we are measuring to what we intended to measure. Exploratory factor analysis has been widely used in medicine to evaluate validity.

Regarding inter-rater reliability, the ICC value of FPI-6 total score was 0.97 and greater than 0.75. Such high ICC value indicated that the inter-rater reliability of FPI-6 in the evaluation of foot posture in people with LBP was excellent. The absence of data on inter-rater reliability of FPI-6 in LBP patients precluded comparison with standard data of this population. Nevertheless, a study examining the inter-rater reliability of FPI-6 in healthy participants with no symptoms reported that the ICC value of FPI-6 total score was 0.81 (greater than 0.75 but lower than our study). It might be affected by large variations between participants and familiarity with the FPI-6. As this study mentioned, the participants were their first subject group to be examined^32^. In addition, all six items showed excellent inter-rater reliability (ICC > 0.75). The ICC values (0.80 to 0.90) were higher than those in a previous study^17^. The first reason might be related to the familiarity with the FPI-6 for raters. The raters in our study completed 40 measurement of FPI-6 before making formal measurement and the data collected was not used, while the raters in previous study had no previous knowledge of this clinical tool. The second reason might be explained by the time interval. The participants in our study were firstly assessed by one rater (J Y), then remained in their positions and were assessed by another rater. While the participants in previous study were assessed at different occasions with 1 h interval. Nevertheless, the ICC value of item 2 was the lowest and its inter-rater reliability was a bit lower compared with the other five items. The result was in accordance with a published study^34^, this indicated that the raters had difficulty in assessing the curves above and below the lateral malleolus and this item may require more training time to distinguish the subtle differences.

In terms of test–retest reliability, the ICC value of FPI-6 total score was 0.95 and all six items ranged between 0.80 and 0.90. These results demonstrated that the test–retest reliability of FPI-6 in the evaluation of foot posture in participants with LBP was excellent. These results were similar to a previous study where the ICC value of FPI-6 total score was 0.985^21^. Notably, the rater in this previous study had experience using the FPI-6 to rate 20 feet and the ICC value for the last 20 feet (ICC = 0.753) examined was higher than the first 20 feet (ICC = 0.985). As mentioned in the previous study, there was an experience or learning effect in administering the FPI-6. Therefore, they recommended that clinical raters have experience using FPI-6 to assess at least 20 feet before using their values for further analysis in order to minimize the inter-rater error. However, another study reported that the ICC value of FPI-6 total-score was 0.69 and test–retest reliability of six items varied from fair to good^35^. Obviously, these results were poor than those in our study. These different findings might be explained by the higher level of experience of the raters in our study. Additionally, the healthy participants (most of them were female) included in that study might also contributed to the different results. Furthermore, studies^35,36^ demonstrated not reliable to moderate retest reliability for older people. The lower reliability for older people may be related to the difficulty in visualizing bony structures. For young children, there is evidence that its inter-rater and test–retest reliably were lower than older children and adults.

SEM and MDC values were used to assess the absolute reliability, which were calculated as SEM = standard deviation×$\surd$1 − ICC and MDC = 1.96 × SEM×$\surd$2^25^. The larger of ICC values, the smaller the SEM value, while the higher of SEM value, the larger of MDC value. A smaller SEM value suggested a better absolute reliability and MDC value represented the minimal amounts of changes need to be considered as a real change and exceed the random errors^37^. The SEM value of FPI-6 total score for inter-rater and test–retest was 0.17 and the MDC value of FPI-6 total score for inter-rater and test–retest was 0.47. Additionally, the SEM value of all six items for inter-rater and test–retest ranged from 0.03 to 0.05 and the MDC of all six items for inter-rater and test–retest ranged from 0.08 to 0.14. These results indicated that the inter-rater and test–retest agreement of the FPI-6 in the evaluation of foot posture in participants with LBP was excellent. The finding was similar to a published study where the results showed excellent inter-rater and test–retest agreement of the FPI-6 in the evaluation of foot posture in healthy adults^17^.

The Bland–Altman plots were used to identify degree of agreement for FPI-6. The plots showed that there was no significant systematic bias for the agreement of FPI-6. The mean difference of FPI-6 total score for inter-rater and test–retest was 0.10 and 0.03, with the lower and upper limits of − 1.09 to 1.29 and − 1.33 to 1.39, respectively. There was little systematic bias of the FPI-6 and the agreement for inter-rater and test–retest was excellent. Studies on the reliability of FPI-6 rarely conducted Bland–Altman analyses, which precludes comparison with published studies. Nevertheless, there is evidence that^38^ the mean difference < 1 represented no systematic bias.

In terms of the validity, the results indicated the good validity of the FPI-6 in the evaluation of LBP. First the EFA was conducted indicating the adequacy of the construct of FPI-6 in our sample. The factor analysis suggested that the fit indices were considered acceptable according to the KMO value (KMO = 0.62) and Bartlett's sphericity test (*P* < 0.01). Two factors were retained based on the principal component analysis and the varimax rotation scheme. Factor loading aimed to demonstrate that how each item contributed the factor formation. The results showed that factor 1 was composed by items 1, 2, 4 and 6; factor 2 by items 3 and 5. In addition, the Spearman’s correlation coefficient was applied to identify the correlation between each item and total score of FPI-6 to complement the factor analysis^30^. The results showed that there was a statistically significant positive correlation between each item and total score of FPI-6. The Spearman’s correlation coefficient of six items in our study were all > 0.3 (*P* value < 0.01). These results were close to a published study where showed the good construct validity of FPI-6 in healthy participants or neuromuscular disease sample^15^.

Sixty limbs of 30 participants were assessed at three different times. There was no difference in classification of feet postures for inter-rater and test–retest (a chi-square test *P*-value = 0.874). When analyzing a total of 60 limbs, only 4 posture classifications changed for the inter-rater and test–retest. The result indicated the reproducibility of the FPI-6 clinically, which was close to a published study^17^. In their study, of a total of 252 limbs, only 11 and 12 posture classifications changed, respectively, for the inter-rater and test–retest. In addition, most of the feet were classified as pronated or highly pronated, which was in accordance with previous studies. foot pronation may cause changes of lower limb biomechanics during walking, which in turn may lead to normal postural changes of pelvis and lumbar vertebrae, finally contributing to LBP^13^. Different types of foot orthoses were used to prevent or treat LBP. This treatment could improve symptoms of LBP through altering abnormal foot posture and influence kinematic posture of lower limb and pelvis^39^. The application of FPI-6 is helpful to understand foot types in patients with LBP, so that appropriate foot orthoses could be used according to the different foot types^10^.

Other devices were also used to investigate the foot posture and function and might be more accurate^7,14^. Nevertheless, previous studies indicated that the accuracy of FPI-6 is consistent with plantar pressure sensor^40^. More importantly, FPI-6 can be used almost everywhere and particularly applied for studies where complex instrumented assessment is unnecessary. Additionally, it could observe and measure foot posture features quickly and easily. The correlation between FPI-6 and plantar pressure sensor on foot posture evaluation in patients with LBP will be further investigated in our future study.

## Limitations

Some limitations of this study should be considered. The volunteers recruited were limited (n = 30), although it reached the minimum numbers of participants required in reliability studies (n = 16). Moreover, although the results showed excellent inter-rater, test–retest reliability and good validity of the FPI-6, all volunteers were recruited from one city (Guangzhou). Thus, Large-scale clinical studies are hence desirable to be conducted in different cities in China. An additional limitation of this study was inadequate refinement of FPI-6 categories. Owing to the nature of the FPI-6 (each of the six criteria has only five possible scores for all foot types), foot types that are not extreme may have less easily categorized characteristics, making selection of the appropriate criterion score less accurate. Therefore, further refinement of the definitions of FPI-6 criterion scores may improve the reliability and validity.

## Conclusion

The inter-rater and test–retest reliability and validity of FPI-6 on people with LBP were proved reliable. It might be considered a reliable and valid adjunctive tool to detect possible changes of foot posture after interventions in patients with LBP.

## Data Availability

All data are available within the manuscript.

## References

[1] Maher C, Underwood M, Buchbinder R (2017). Non-specific low back pain. Lancet (London, England).

[2] Alford VM (2015). The use of the International Classification of Functioning, Disability and Health to understand the health and functioning experiences of people with chronic conditions from the person perspective: A systematic review. Disabil. Rehabil..

[3] Tagliaferri SD (2020). Domains of chronic low back pain and assessing treatment effectiveness: A clinical perspective. Pain Pract..

[4] Cieza A (2021). Global estimates of the need for rehabilitation based on the Global Burden of Disease study 2019: a systematic analysis for the Global Burden of Disease Study 2019. Lancet (London, England).

[5] Knezevic NN, Candido KD, Vlaeyen JWS, Van Zundert J, Cohen SP (2021). Low back pain. Lancet (London, England).

[6] Hartvigsen J (2018). What low back pain is and why we need to pay attention. Lancet (London, England).

[7] Menz HB, Dufour AB, Riskowski JL, Hillstrom HJ, Hannan MT (2013). Foot posture, foot function and low back pain: The Framingham Foot Study. Rheumatology (Oxford).

[8] Lee SW (2018). Footwear-generated dynamic biomechanical manipulation and perturbation training for chronic nonspecific low back pain. PM R.

[9] Balasundaram AP, Choudhury D (2018). Association between hyper-pronated foot and the degree of severity of disability in patients with non-specific low back pain. J. Bodyw. Mov. Ther..

[10] Sadler S, Spink M, Cassidy S, Chuter V (2018). Prefabricated foot orthoses compared to a placebo intervention for the treatment of chronic nonspecific low back pain: A study protocol for a randomised controlled trial. J. Foot Ankle Res..

[11] Castro-Méndez A, Munuera PV, Albornoz-Cabello M (2013). The short-term effect of custom-made foot orthoses in subjects with excessive foot pronation and lower back pain: A randomized, double-blinded, clinical trial. Prosthet. Orthot. Int..

[12] Betsch M (2011). Influence of foot positions on the spine and pelvis. Arthritis Care Res..

[13] Kendall JC, Bird AR, Azari MF (2014). Foot posture, leg length discrepancy and low back pain-their relationship and clinical management using foot orthoses—An overview. Foot (Edinb.).

[14] Zammit GV, Menz HB, Munteanu SE (2010). Reliability of the TekScan MatScan(R) system for the measurement of plantar forces and pressures during barefoot level walking in healthy adults. J. Foot Ankle Res..

[15] Keenan AM, Redmond AC, Horton M, Conaghan PG, Tennant A (2007). The Foot Posture Index: Rasch analysis of a novel, foot-specific outcome measure. Arch. Phys. Med. Rehabil..

[16] Redmond AC, Crane YZ, Menz HB (2008). Normative values for the Foot Posture Index. J. Foot Ankle Res..

[17] Martinez BR, Oliveira JC, Vieira K, Yi LC (2021). Translation, cross-cultural adaptation, and reliability of the Foot Posture Index (FPI-6)—Brazilian version. Physiother. Theory Pract..

[18] Buldt AK (2015). Are clinical measures of foot posture and mobility associated with foot kinematics when walking?. J. Foot Ankle Res..

[19] Terada M, Wittwer AM, Gribble PA (2014). Intra-rater and inter-rater reliability of the five image-based criteria of the foot posture index-6. Int. J. Sports Phys. Ther..

[20] Zou GY (2012). Sample size formulas for estimating intraclass correlation coefficients with precision and assurance. Stat. Med..

[21] Cornwall MW, McPoil TG, Lebec M, Vicenzino B, Wilson J (2008). Reliability of the modified Foot Posture Index. J. Am. Podiatr. Med. Assoc..

[22] Redmond AC, Crosbie J, Ouvrier RA (2006). Development and validation of a novel rating system for scoring standing foot posture: The Foot Posture Index. Clin. Biomech. (Bristol, Avon).

[23] Koo TK, Li MY (2016). A guideline of selecting and reporting intraclass correlation coefficients for reliability research. J. Chiropr. Med..

[24] van Geel N, Passeron T, Wolkerstorfer A, Speeckaert R, Ezzedine K (2020). Reliability and validity of the Vitiligo Signs of Activity Score (VSAS). Br. J. Dermatol..

[25] Silva AG (2019). Inter-rater reliability, standard error of measurement and minimal detectable change of the 12-item WHODAS 2.0 and four performance tests in institutionalized ambulatory older adults. Disabil. Rehabil..

[26] Chen G (2019). Reliability of a portable device for quantifying tone and stiffness of quadriceps femoris and patellar tendon at different knee flexion angles. PLoS ONE.

[27] Manera M (2021). Exploratory factor analysis of rainbow trout serum chemistry variables. Int. J. Environ. Res. Publ. Health.

[28] Han JX, Wang K (2021). Health Informatics on adolescents smoking based on the Miryoku engineering analysis framework. J. Healthc. Eng..

[29] So H (2021). Development and validation of a food literacy assessment tool for community-dwelling elderly people. Int. J. Environ. Res. Publ. Health.

[30] Vignola RC, Tucci AM (2014). Adaptation and validation of the depression, anxiety and stress scale (DASS) to Brazilian Portuguese. J. Affect. Disord..

[31] Koo HC (2020). Development, validity and reproducibility of a whole grain food frequency questionnaire in Malaysian children. Nutr. J..

[32] Evans AM, Copper AW, Scharfbillig RW, Scutter SD, Williams MT (2003). Reliability of the foot posture index and traditional measures of foot position. J. Am. Podiatr. Med. Assoc..

[33] Hallit S (2021). Adaptation of the Young Adults' Cigarette Dependence (YACD) Scale for the development and validation of the Adolescent Cigarette Dependence Scale (ACDS). Environ. Sci. Pollut. Res. Int..

[34] McLaughlin P, Vaughan B, Shanahan J, Martin J, Linger G (2016). Inexperienced examiners and the Foot Posture Index: A reliability study. Man. Ther..

[35] Aquino MRC, Avelar BS, Silva PL, Ocarino JM, Resende RA (2018). Reliability of Foot Posture Index individual and total scores for adults and older adults. Musculoskeletal Sci. Pract..

[36] Menz HB, Munteanu SE (2005). Validity of 3 clinical techniques for the measurement of static foot posture in older people. J. Orthop. Sports Phys. Ther..

[37] Liao WW (2020). Test–retest reliability and minimal detectable change of the Contextual Memory Test in older adults with and without mild cognitive impairment. PLoS ONE.

[38] Fernandes R, Armada-da-Silva P, Pool-Goudzwaard AL, Moniz-Pereira V, Veloso AP (2015). Test–retest reliability and minimal detectable change of three-dimensional gait analysis in chronic low back pain patients. Gait Posture.

[39] Castro-Méndez A (2021). Custom-made foot orthoses as non-specific chronic low back pain and pronated foot treatment. Int. J. Environ. Res. Publ. Health.

[40] Teyhen DS (2011). Static foot posture associated with dynamic plantar pressure parameters. J. Orthop. Sports Phys. Ther..

